# Real-World Effectiveness of Second-Line Therapies in Advanced Non-Small Cell Lung Cancer: Insights From Propensity-Weighted Comparative Analyses of Longitudinal EHR Data

**DOI:** 10.1016/j.cllc.2025.09.010

**Published:** 2025-09-26

**Authors:** Gary C.N. Hettinger, Qi Long, Ravi B. Parikh

**Affiliations:** 1 Department of Population Health, New York University Grossman School of Medicine, New York, NY; 2 Department of Biostatistics, Epidemiology, and Informatics, University of Pennsylvania Perelman School of Medicine, Philadelphia, PA; 3 Abramson Cancer Center, University of Pennsylvania, Philadelphia, PA; 4 Department of Hematology and Medical Oncology, Emory University School of Medicine, Atlanta, GA

**Keywords:** Biomarker, Causal inference, Chemotherapy, Immunotherapy, Targeted therapy

## Abstract

This study evaluated real-world effectiveness of second-line therapies in 3,454 patients with advanced non-small cell lung cancer using longitudinal electronic health record-derived data. Propensity-weighted analyses revealed meaningful survival differences across second-line treatments among patients progressing on the same first-line regimen category. These findings emphasize the impact of second-line treatment decisions and the importance of real-world evidence in guiding care.

**Background:**

While substantial research has focused on first-line (1L) therapies for advanced-stage non-small cell lung cancer (aNSCLC), less is known about the effectiveness of second-line (2L) treatments following disease progression on 1L. The poor prognosis after 1L progression, limited guidance for 2L treatment, and constraints of clinical trials in addressing these questions underscore the need for real-world comparative effectiveness research.

**Methods:**

We conducted a retrospective analysis to compare real-world overall survival (rwOS) associated with 2L treatments among patients with disease progression during 1L. Eligible patients were selected from Flatiron Health’s nationwide electronic health record (EHR)-derived de-identified database. 1L and 2L treatments were categorized as chemotherapy-only (Chemo-alone), combination chemo- and immunotherapy (Chemo + Immuno), immunotherapy-only (IO-alone), targeted-alone therapy, or targeted-plus therapy. We used inverse probability of treatment weighting to adjust for extensive static and longitudinal patient-level confounding and compared rwOS using Kaplan-Meier curves and restricted mean survival time (RMST).

**Results:**

Among patients previously treated with 1L Targeted therapy, those receiving 2L Targeted-alone therapy had longer life expectancies than those receiving a nontargeted (ΔRMST-36 + 2.61 months) or Targeted-plus regimen (ΔRMST-36 + 3.11 months). For patients progressing on 1L Chemo + Immuno, 2L Chemo + Immuno outperformed Chemo-alone (ΔRMST-36 + 2.98 months). Among patients progressing ≥1 year after initiating 1L IO-alone, 2L Chemo + Immuno again showed better life expectancy than Chemo-alone (ΔRMST-36 + 5.70 months).

**Conclusions:**

Longitudinal real-world data can enhance assessment of 2L therapy effectiveness. After confounder adjustment, we found clinically meaningful survival differences by 2L choice. These findings underscore the importance of 2L treatment selection and the need for prospective validation.

## Introduction

The 5-year survival rate for patients diagnosed with advanced stage non-small cell lung cancer (aNSCLC) is currently 9%.^1–3^ Nevertheless, recent approvals of novel first-line therapy (1L) treatments suggest promising prospects for improved outcomes among these patients.^4–6^

When the disease progresses on 1L, clinicians face the critical challenge of selecting an appropriate second-line (2L) regimen, a setting in which median survival is often estimated to be less than 1 year.^7–10^ Although numerous studies have suggested that the decision regarding 2L treatment can substantially impact outcomes, there is a paucity of phase III randomized control trial (RCT) data for current 2L options because few such trials have been conducted in the recent treatment era.^7–29^ National Comprehensive Cancer Network (NCCN) guidelines offer some insights into preferred 2L choices, but these recommendations are generally not grounded in RCT data across comprehensive comparisons.^5^ Even when clinical trials are conducted, their findings often face limitations in generalizability due to patient selection biases arising from enrollment requirements and health disparities, provider selection biases in that trials may be conducted at nonrepresentative centers, and the controlled conditions inherent to trial environments.^30^ Meanwhile, real-world data (RWD) studies, while valuable for informing comparative effectiveness on representative populations, often face limitations such as confounding by indication and a lack of thorough health information for appropriate adjustment.^31, 32^ However, recent advancements in the availability of detailed RWD and covariate-balancing propensity score methodologies for survival analyses may alleviate these limitations, as evidenced by prior findings.^33^

This retrospective cohort study aims to leverage rich longitudinal patient-level RWD and propensity score methods to accurately compare the effectiveness of current 2L therapies on real-world survival outcomes. Specifically, we compare the effectiveness of 5 modern 2L regimens – chemotherapy-only (Chemo-alone), immunotherapy-only (IO-alone), combination chemo- and immunotherapy (Chemo + Immuno), targeted-alone, and targeted-plus therapy – for 3 representative patient cohorts – those receiving a 1L Targeted therapy, Chemo + Immuno, and IO-alone regimen. In addition to assessing how survival outcomes vary with different 2L treatment decisions, our study underscores the critical role of real-world evidence in evaluating therapeutic effectiveness beyond aNSCLC clinical trials, illustrates the use of EHR data in this context, and highlights the need for prospective studies to further refine 2L treatment strategies in aNSCLC.

## Materials and Methods

### Study Sample

**Figure 1:**
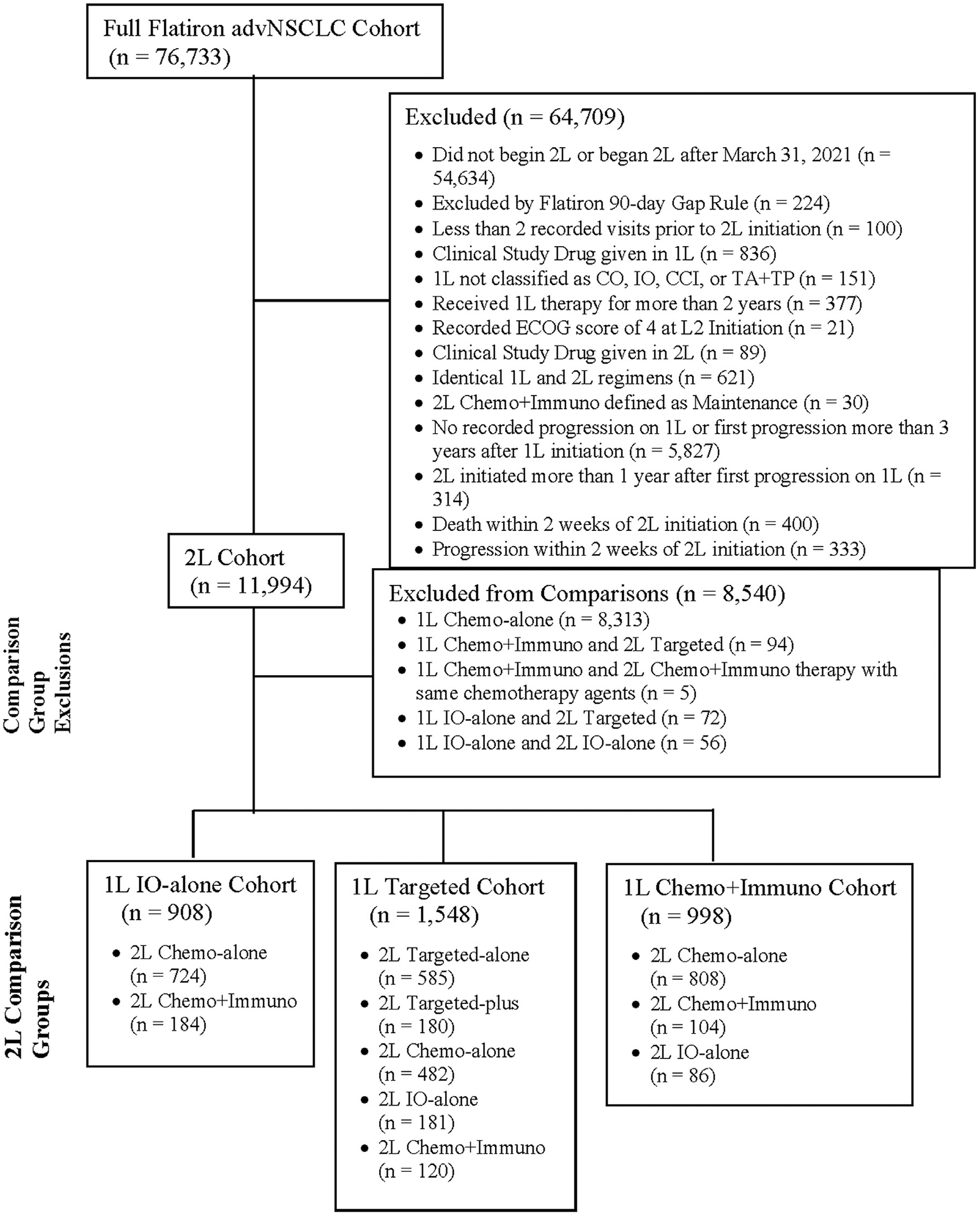
Consort diagram for NSCLC 2L treatment effectiveness comparisons.

This study used the nationwide Flatiron Health electronic health record (EHR)-derived de-identified database. This database is a longitudinal database, comprising de-identified patient-level structured and unstructured data, curated via technology-enabled abstraction.^34, 35^ During the study period, the de-identified data originated from approximately 280 cancer clinics (~800 sites of care), most being community oncology settings. The study included patients 18 years or older first diagnosed with an advanced stage of NSCLC after January 1, 2011, who initiated systemic 1L and 2L therapy regimens prior to March 31, 2021 (*n* = 22,099). Patients without an identified progression within 3 years of 1L initiation and prior to 2L initiation were not considered (*n* = 5827). Progressions were defined as distinct episodes in the patient journey at which the treating clinician concluded that there was spread or worsening in the disease of interest and identified through a multi-step abstraction process.^36^ We excluded patients with a recorded Eastern Cooperative Oncology Group (ECOG) performance status>3 at the time of 2L initiation (*n* = 21), as these patients are generally poor candidates for chemotherapy. Additionally, we excluded patients who progressed or died within 14 days of 2L initiation (*n* = 400), as treatment outcomes in this scenario are unlikely driven by choice of 2L therapy. We also excluded patients who had no evidence of structured EHR activity within 90 days of advanced stage diagnosis (*n* = 224), had less than 2 recorded visits in the database between advanced diagnosis date and 2L initiation (*n* = 100), or received 1L or 2L agents as part of a randomized clinical trial as these patients may have misclassified lines of therapy (*n* = 925). We also excluded patients who received medication for their 1L regimen for more than 2 years (*n* = 377), received an identical regimen in 1L and 2L (*n* = 621), received maintenance 2L defined by 1L regimen (*n* = 30), or initiated 2L greater than 1 year after their first progression on 1L (*n* = 314) as these patients may have a less severe progression between lines and/or do not follow standard treatment guidance (Figure 1).

### Categorization of 1L and 2L Treatment Strategies

Using oncologist-defined, rule-based lines of therapy, we then identified broad categories of therapeutic options by following NCCN guidelines for nonmaintenance first- and second-line systemic therapy.^5^ These lines were denoted as Chemo-alone, IO-alone, Chemo + Immuno, or Targeted regimens. For 2L lines, we further delineated between Targeted-alone and Targeted-plus regimens. A summary of classification rules is presented in Supplemental Figure 1 and specific line classifications are presented in Supplemental Tables 1,2A and 2B. Patients receiving a 1L Chemo-alone, IO-alone, Chemo + Immuno, or Targeted regimen were considered in the overall cohort used for imputation models. We then stratified by 1L regimen to compare the efficacy of relevant 2L regimens (Table 1).

### Outcomes

The primary outcome was real-world overall survival (rwOS) from 2L initiation to date of death.^37^ Follow-up was terminated at the last date at which there is EHR evidence that a patient is alive using data either confirmative of a patient being seen in Flatiron Health’s network or providing evidence of clinical confirmation of treatment receipt. A secondary outcome was real-world Progression-Free Survival (rwPFS) from 2L initiation to first tumor progression date or date of death, whichever occurs first.^38^ Here, follow-up was terminated using the date of last clinic note and real-world progressions were abstracted and defined as distinct episodes in the patient journey at which the treating clinician concludes that there has been spread or worsening in the disease of interest.

### Covariates

We selected 12 confounding variables for our primary covariate-balanced analyses, based on their associations with aNSCLC treatment decisions and survival, as well as their inclusion in prior aNSCLC studies: rwPFS from 1L initiation, insurance category (Medicaid, Medicare or other federal insurance, commercial, or other), de novo vs. recurrent metastatic disease at diagnosis (yes/no), aNSCLC diagnosis year (pre-2016, 2016–2018, post-2018), history of smoking (yes/no), age at 2L initiation, gender (M/F), ECOG score at 2L initiation, change in ECOG score during 1L, number of newly recorded comorbidities during 1L, race (White/Not White), and the type of practice where the patient received 2L (academic/community).^22, 28, 33^ For the 1L Chemo + Immuno and IO-alone cohorts, we also included an indicator for tumor histology (squamous/nonsquamous) but merged the pre-2016 and 2016–2018 diagnosis year categories due to limited pre-2016 data for certain 2L groups. For sensitivity analyses, we defined 2 more variable sets – an Extended set and a Comprehensive set – which augment the original confounder set by including additional longitudinal labs, vital measurements, and other potential confounders. These sets are described further in Supplemental Table 3.

### Statistical Analysis

For each 1L cohort, we estimated and compared inverse probability of treatment-weighted (IPTW) Kaplan-Meier curves and restricted mean survival time at 12 (RMST-12) and 36 (RMST-36) months for each 2L category.^39–42^ RMST is defined as the area under the survival curve up to a specific time point and was chosen for ease of interpretability – the life expectancy during the period from 2L initiation to the follow-up time – and comparison – analyses do not rely on the proportional hazards assumption which appear violated for certain comparisons.^43^ As many covariates had incomplete information in our dataset, we first created 60 imputations using chained equations where missing data were imputed based on the Comprehensive variable set with minimum proportion of usable cases of 0.2 and minimum correlation of 0.05.^44, 45^ Then, we estimated propensity score weights for estimating the average treatment effect for each imputed dataset using multinomial logistic regression models for the probability of receiving a 2L regimen within each 1L cohort. We assessed postweighting balance by first taking the average standardized mean difference (SMD) per 2L comparison per 1L cohort across imputations. In each cohort, we then calculated the average and maximum SMD between 2L pairs and the fraction of covariates considered imbalanced in any pair (SMD < 0.1). To assess 2L performance, we then calculated survival metrics and pooled results across imputations using Rubin’s rules.^46^

To explore potential effect heterogeneity and mechanisms, we repeated these analyses on subgroups defined by rwPFS from 1L initiation (greater/less than 1 year), 2L ECOG score (0–1, 2–3), and biomarker status (any/no recorded positive biomarker test result prior to 2L initiation). Finally, we assessed the sensitivity of our conclusions after extending the exclusion period window length from 14 to 28 days (*n* = 375) and under various weighting approaches – energy-balancing weights using the Extended variable set truncated at the fifth and 95th percentiles, weights from a multinomial LASSO model using the Comprehensive variable set truncated at the first and 99th percentiles, and without using any weights.^47, 48^ Statistical analyses were performed in R version 4.0.2 (R Group for Statistical Computing).

## Results

### Demographic Characteristics

Of the 76,733 aNSCLC patients, 11,994 met all eligibility criteria (Figure 1). Most eligible patients received 2L Chemo-alone regimens until IO-alone regimen usage peaked in 2016 (Supplemental Figure 2). Of these 11,994 patients, 5894 (49%) received a 2L Chemo-alone regimen, 659 (6%) received a 2L Chemo + Immuno regimen, 3875 (32%) received a 2L IO-alone regimen, 1266 (11%) received a 2L Targeted-alone regimen, and 300 (3%) received a 2L Targeted-plus regimen. Median age at 2L initiation was 68 years (IQR 61–75) (Table 2). Most patients were Male (*n* = 6254 [52%]), Non-Hispanic White (*n* = 8269 [73%]), treated in a community-based oncology practice (*n* = 10,753 [90%]), had a history of smoking (*n* = 10,233 [86%]), and a nonsquamous histology (*n* = 8603 [75%]). Patients were covered by commercial insurance (*n* = 2758 [26%]), Medicaid (*n* = 1405 [13%]), other governmental insurance (*n* = 4568 [42%]), and other insurance (*n* = 2025 [19%]). Four primary covariates had more than 5% missingness: Insurance (*n* = 1238 [10%]), ECOG score (*n* = 2418 [20%]), ECOG change (*n* = 3574 [30%]), and Race (*n* = 650 [5%]). The median time for patients from 1L initiation to first real-world progression was 5.06 months (95% CI [4.96, 5.19]). From 2L initiation, the median rwOS was 8.38 months (95% CI [8.18, 8.61]) and the median rwPFS was 3.45 months (95% CI [3.35, 3.55]). Demographic characteristics for separate 1L therapy categories are presented stratified by 2L treatment group in Supplemental Tables 4–6.

### Comparative Effectiveness of 2L by 1L Therapy Category

#### 1L Targeted Therapy.

**Figure 2:**
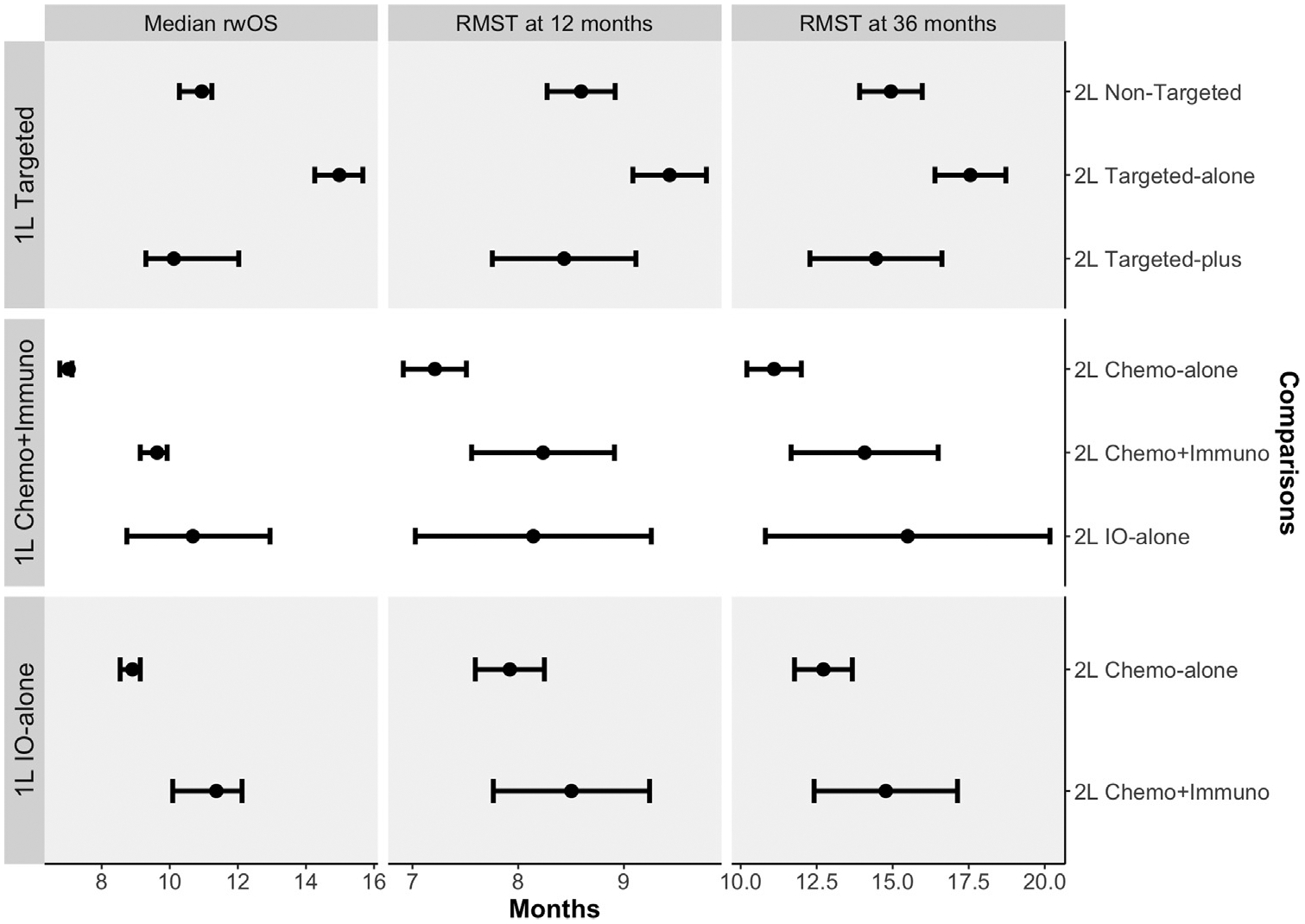
Inverse Probability Weighted (IPW)-adjusted summaries of real-world overall survival (rwOS) stratified by first-line (1L) and second-line (2L) treatment decisions. RMST = restricted mean survival time.

**Figure 3:**
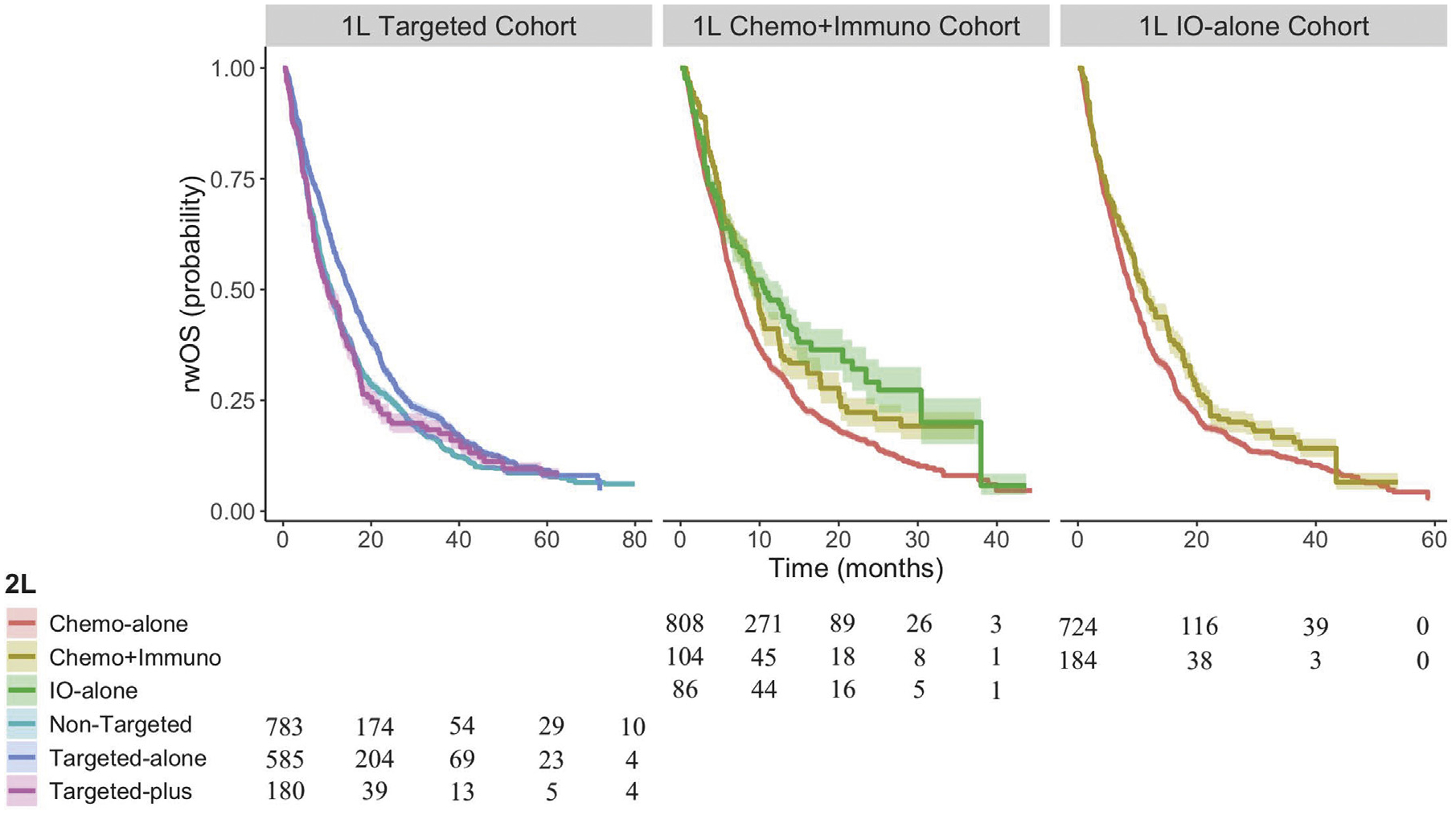
Inverse probability weighted (IPW)-adjusted Kaplan Meier curves of real-world overall survival (rwOS) stratified by second-line (2L) treatment choice.

1548 eligible patients underwent 1L targeted therapies, among whom 783 transitioned to a nontargeted regimen in 2L (RMST-36 14.94 months (95% CI [13.91, 15.97]), 585 to a targeted-alone regimen (RMST-36 17.55 months (95% CI [16.39, 18.72]), and 180 to a targeted-plus regimen (RMST-36 14.45 months (95% CI [12.27, 16.62])) (Figure 2). After weighting, all measured confounders were considered balanced between the 2L groups (Supplemental Table 7). Adjusted Kaplan-Meier curves are shown in Figure 3.

2L targeted-alone regimens were associated with a higher rwOS than nontargeted regimens (RMST-36 difference +2.61 months [95% CI {+1.06, +4.17}]) and targeted-plus regimens (RMST-36 difference +3.11 months [95% CI {+0.64, +5.57}]) (Table 3). These differences in life expectancy were also statistically significant at 12 months (RMST-12 difference +0.84 months [95% CI {+0.36, +1.31}], +1.00 months [95% CI {+0.24, +1.76}]) and were driven by patients with an ECOG score of 0–1 (RMST-36 difference +3.16 months [95% CI {+1.16, +5.16}], +3.34 months [95% CI {+0.16, +6.51}]) rather than 2–3 (RMST-36 difference +1.35 months [95% CI {−1.99, +4.67}], −0.87 months [95% CI {−8.70, +6.95}]) (Supplemental Figure 3). The differences in RMST-12 and RMST-36 between 2L nontargeted and targeted-plus regimens were not significant (RMST-12 difference +0.16 [95% CI {−0.59, +0.91}], RMST-36 difference +0.49 [95% CI {−1.91, +2.90}]). Conclusions were consistent for rwPFS (Supplemental Figures 4–5).

#### 1L Chemo + Immuno Therapy.

Among 998 eligible patients who underwent 1L Chemo + Immuno regimens, 808 received a chemo-alone regimen in 2L (RMST-36 11.10 months (95% CI [10.20, 11.99]), 104 a Chemo + Immuno regimen (RMST-36 14.08 months (95% CI [11.66, 16.49]), and 86 an IO-alone regimen (RMST-36 15.49 months (95% CI [10.81, 20.17]) (Figure 2). After weighting, all confounders were considered balanced between the 2L groups except for smoking history (SMD 0.15) (Supplemental Table 7).

2L Chemo + Immuno regimens were associated with a higher rwOS (RMST-12 difference +1.02 months (95% CI [+0.28, +1.76]), RMST-36 difference +2.98 months [95% CI [+0.40, +5.56]) than chemo-alone regimens (Table 3). 2L IO-alone regimens demonstrated superior rwOS compared to Chemo-alone regimens, but with high uncertainty (RMST-36 difference +4.39 months [95% CI {−0.37, +9.16}]). This difference is substantially lower for slow-progressing patients (1L rwPFS > 1 year) (RMST-36 difference +0.33 months [95% CI {−4.78, +5.44}]) and those without a recorded mutation (RMST-36 difference +1.60 months [95% CI {−4.01, +7.20}]) which, in addition to the few patients receiving 2L IO-alone regimens, may explain the wide confidence intervals (Supplemental Figure 3). RMST differences were not significantly different between 2L IO-alone and Chemo + Immuno regimens (RMST-36 difference +1.42 months [95% CI {−3.85, +6.68}]). RMST comparisons for rwPFS were mostly statistically insignificant but were highest for 2L Chemo + Immuno regimens (Supplemental Figures 4, 6).

#### 1L IO-alone Therapy.

Among 908 eligible patients who underwent 1L IO-alone regimens, 724 received a chemo-alone regimen in 2L (RMST-36 12.72 months (95% CI [11.77, 13.67]) and 184 a Chemo + Immuno regimen (RMST-36 14.77 months (95% CI [12.41, 17.13]) (Figure 2). After weighting, all confounders were considered balanced (Supplemental Table 7). While rwOS was higher on 2L Chemo + Immuno regimens than Chemo-alone regimens, these associations were not statistically significant (RMST-36 difference +2.05 months [95% CI {−0.49, +4.59}]) (Table 3). However, the difference was substantially higher among slow-progressing patients (RMST-36 difference +5.70 [95% CI {+0.67, +10.72}]) (Supplemental Figure 3). Estimated effects on rwPFS are small and statistically insignificant (Supplemental Figures 4, 7).

### Sensitivity Analyses

Conclusions were consistent across exclusion periods and confounding adjustment approaches, with a few exceptions. Notably, some comparisons were significant without any confounding adjustment, and the comparison between 2L Chemo + Immuno and Chemo-alone among 1L Chemo + Immuno patients showed attenuated effects when using energy-balancing weights with the Extended variable set (Supplemental Tables 8–10). While conclusions were largely consistent, the magnitudes of differences varied based on the weighting approach and RMST-36 differences were up to 51% higher (+1.34 months) prior to weighting and 31% lower (−1.33 months) when using energy balancing weights derived from the Extended variable set relative to our primary results, reflecting the important role of confounder adjustment.

## Discussion

This retrospective real-world analysis compared survival outcomes between patients with aNSCLC who had received different second-line treatments after progressing on their first line of advanced stage treatment. After adjusting for past treatment history and potential confounders, we found substantial differences in survival outcomes among patients according to the second-line treatment received.

For patients receiving a first-line targeted regimen, we found evidence of better survival with a second-line targeted-alone regimen than a second-line nontargeted or targeted-plus regimen. Previous works have found mixed results regarding the relative effectiveness of different second-line targeted strategies following a first-line chemotherapy regimen. Looking at nonsquamous patients, Lee et al.^14^ and Dittrich et al.^15^ found that the targeted-plus regimen of erlotinib and pemetrexed outperformed both drugs in isolation in terms of survival, despite an increase in toxicity. Qi et al. found improved second-line performance by combining targeted drugs instead of single-agent erlotinib in the second line.^23^ It is possible our findings differ from these results due to differences in patient treatment sensitivity as our cohort had progressed on a first-line targeted, not chemotherapy, regimen. Notably, the AURA3 trial demonstrated superior progression-free survival with osimertinib compared to platinum-based chemotherapy in patients who progressed on prior EGFR TKI therapy^49^ These differences may also result from our inclusion of patients with a smoking history (unlike Lee et al.^14^), higher ECOG scores, different predictive biomarkers, and over a range of treatment options in each category, highlighting the importance of real-world cohorts for projecting real-world effectiveness. For example, Kim et al.^16^ found no benefit of adding cetuximab to second-line chemotherapy regimens and Kim et al.^21^ found that superiority of second-line pemetrexed (chemo-alone) and gefitinib (targeted-alone) regimens depended on EGFR status. Additionally, clinicians may have used unmeasured information such as baseline patient differences or disease burden at progression when making treatment decisions, which could partially explain the observed differences in rwOS between the targeted-alone and targeted-plus groups. We did see some improvement for second-line targeted-plus regimens relative to nontargeted regimens in unweighted subgroup analyses for patients initiating second-line treatment after 2016, so it is possible our conclusions may change with more follow-up time.

For patients receiving a first-line Chemo + Immuno regimen, we found evidence of better survival with a second-line Chemo + Immuno regimen than a second-line chemo-alone regimen. While second-line chemo-alone regimens have been shown to improve survival over palliative care for those progressing on chemo-only regimens,^26^ Auclin et al.^7^ noted only modest activity for second-line chemo-alone regimens among patients progressing on first-line Chemo + Immuno and Bazhenova et al.^8^ found higher survival rates for patients on 2L immunotherapy regimens compared to Chemo-alone. Garon et al. found second-line ramucirumab and docetaxel improved upon docetaxel alone for patients progressing on a first-line chemo-only regimen.^17^ The combination of toripalimab and chemotherapy has also been found to improve performance over chemo-alone regimens among patients progressing on a first-line chemo-alone or targeted regimen.^19, 20^ Our conclusions provide further evidence for patients progressing on first-line Chemo + Immuno regimens, a prominent first-line option in current standard-of-care that has been previously understudied in phase III trials. We also found evidence for improved performance of second-line Chemo + Immuno over chemo-alone therapy among patients progressing on first-line Chemo + Immuno regimen or after a year on a first-line IO-alone regimen, suggesting future patient subsets that may benefit. Recent trials have explored the efficacy of second-line ramucirumab plus pembrolizumab (a targeted-plus regimen) for patients progressing on first-line Chemo + Immuno regimens. While the Lung-MAP S1800A study found better outcomes on ramucirumab plus pembrolizumab compared to standard-of-care, the ongoing PRAGMATICA-Lung trial did not find significant differences in interim analyses.^50, 51^ Future studies should continue to evaluate these targeted-plus regimens as a further benefit as additional real-world data on these options emerge.

To our knowledge, this is the first study to thoroughly investigate the effectiveness of clinically-relevant second-line treatment decisions on a national real-world cohort. Previous works have cited the need to understand real-world effectiveness of NSCLC treatments outside of the clinical trial setting where participating patients and medical centers may be nonrepresentative.^31, 52^ Additionally, we were able to include a rich dataset with patient-level information with the proven ability to mimic randomized trial results under robust confounding adjustment.^33^ Confounder adjustment was highly influential in our dataset, reducing unadjusted differences by up to 51%.

There are several limitations to this analysis. First, the Flatiron Health database is EHR-derived and subject to data entry errors and missing data. Incomplete medical histories may have occurred owing to the database’s inability to capture health visits and treatments that occurred outside of the Flatiron Health network. Incomplete data may therefore reduce our ability to properly adjust for missing data and confounding. We tried to limit the effect of these 2 features of observational studies by conducting multiple imputation analyses and adjusting for a rich set of confounding information, but there may still be unmeasured factors influencing missingness, treatment decisions, and survival. Second, incomplete data capture was especially important for use of predictive biomarkers in our analyses. Previous works have highlighted the importance of such biomarkers in NSCLC treatment efficacy and may explain some of the differences between our findings and those of specific clinical trials.^4, 53, 54^ Notably, resistance mutations, which were incompletely captured, play a substantial role in 2L treatment decisions, particularly for patients with tumors harboring driver mutations. We tried to minimize the influence of biomarkers by conditioning on first-line treatment group as a proxy for certain biomarker eligibility. However, this approach may not fully address imbalances between comparison groups on these factors. To address this limitation, we performed subgroup analyses based on recorded mutations, which demonstrated similar associations as our primary analyses, although these subgroups may still lack the granularity needed to capture all relevant biomarker-related nuances. Third, the overall patient population in the database may not be fully representative of the overall population of patients with aNSCLC in the United States. The patient population is limited by Flatiron Health network participation and skewed heavily towards patients in community-based practice. Relatedly, certain treatment regimens, particularly more recent advances, may be over- or underrepresented due to variation in practice patterns and the timing of patient treatment, which requires adequate follow-up for inclusion.

Future research should aim to validate these findings through prospective data collection, which could provide valuable insights into the effects of recent therapeutic advances and treatment strategies. A key area for further exploration is the role of biomarkers, which remain inadequately captured in this dataset and exhibit missingness influenced by shifting clinical practices over time. Studies focusing on patient populations with comprehensive biomarker documentation could provide critical insights into individualized treatment strategies. Finally, leveraging longitudinal RWD on patient health measures from the 1L setting may ultimately enhance 2L risk predictions and facilitate more precise, personalized treatment decisions.

In conclusion, understanding optimal second-line treatment decisions is of great value in aNSCLC care. Prognosis for patients after their aNSCLC progresses is extremely poor and standard-of-care guidelines for first-line treatments may no longer apply when the first choice fails. This study provides data relevant for clinicians and patients balancing 2L treatment decisions with other considerations like quality-of-life on each treatment as well as researchers working on treatment solutions. Our study offers both evidence and a methodological framework to support further evaluation of second-line treatment decisions in aNSCLC.

## Supplementary Material

1

## Figures and Tables

**Table 1:** 1L and 2L Therapy Categories Considered for Comparison

1L Category	2L Categories Compared
Targeted (*n* = 1548)	Nontargeted (*n* = 783), targeted-alone (*n* = 585), targeted-plus (*n* = 180)
Chemo + Immuno (*n* = 1028)	Chemo-alone (*n* = 808), Chemo + Immuno (*n* = 104), IO-alone (*n* = 86)
IO-alone (*n* = 908)	Chemo-alone (*n* = 724), Chemo + Immuno (*n* = 184)

**Table 2:** Patient Characteristics Prior to Second-Line (2L) Treatment Initiation Stratified by First-Line (1L) Treatment Regimen

Patient Characteristic	1L Targeted (*n* = 1548)	1L Chemo + Immuno (*n* = 998)	1L IO-alone (*n* = 908)
2L treatment regimen, No. (%)			
Chemo-alone	783 (50.6)	808 (81.0)	724 (79.7)
Chemo + Immuno		104 (10.4)	184 (20.3)
IO-alone		86 (8.6)	0 (0.0)
Targeted-alone	585 (37.8)	0 (0.0)	0 (0.0)
Targeted-plus	180 (11.6)	0 (0.0)	0 (0.0)
Advanced stage at initial diagnosis, No. (%)	1206 (77.9)	811 (81.3)	447 (49.2)
Age at 2L initiation, mean (SD)	66.86 (10.70)	67.09 (9.16)	69.49 (9.48)
Change in ECOG score during 1L, Mean (SD)	0.14 (0.80)	0.22 (0.69)	0.23 (0.72)
ECOG score at 2L Initiation, No. (%)			
0	342 (22.1)	242 (24.2)	190 (20.9)
1	575 (37.1)	437 (43.8)	410 (45.2)
2–3	260 (16.8)	209 (20.9)	190 (20.9)
Unknown	371 (24.0)	110 (11.0)	118 (13.0)
Insurance type, No. (%)			
Commercial	403 (26.0)	254 (25.5)	197 (21.7)
Ever on medicaid	157 (10.1)	127 (12.7)	110 (12.1)
Government	579 (37.4)	371 (37.2)	388 (42.7)
Other	259 (16.7)	198 (19.8)	177 (19.5)
Unknown	150 (9.7)	48 (4.8)	36 (4.0)
Male, No. (%)	588 (38.0)	554 (55.5)	464 (51.1)
Number of new diagnoses during 1L, Mean (SD)	0.73 (1.06)	0.88 (1.17)	0.62 (0.93)
Smoking history, No. (%)			
No	737 (47.6)	91 (9.1)	73 (8.0)
Yes	798 (51.6)	907 (90.9)	835 (92.0)
Unknown	13 (0.8)	0 (0.0)	0 (0.0)
Squamous histology at initial diagnosis, no. (%)			
No	1402 (90.6)	774 (77.6)	563 (62.0)
Yes	113 (7.3)	188 (18.8)	313 (34.5)
Unknown	33 (2.1)	36 (3.6)	32 (3.5)
Time until progression on 1L, Mean (SD)	7.98 (5.36)	6.29 (4.69)	5.34 (4.75)
Treated at community practice, No. (%)	1290 (83.3)	915 (91.7)	812 (89.4)
White, no. (%)			
No	494 (31.9)	257 (25.8)	208 (22.9)
Yes	955 (61.7)	682 (68.3)	651 (71.7)
Unknown	99 (6.4)	59 (5.9)	49 (5.4)
Year of advanced stage diagnosis, No. (%)			
<2016	797 (51.5)	6 (0.6)	112 (12.3)
2016–2019	595 (38.4)	464 (46.5)	575 (63.3)
2019+	156 (10.1)	528 (52.9)	221 (24.3)

**Table 3:** Inverse Probability Weighted (IPW)-Adjusted Comparisons of Real-World Overall Survival (rwOS) Using Restricted Mean Survival Time (RMST) Estimates Across Second-Line (2L) Treatment Choices

1L Treatment	2L Treatment Comparison	ΔRMST-12 (Months)	ΔRMST-36 (Months)
Targeted	Targeted-alone vs. nontargeted	0.84 (0.36, 1.31)^a^	2.61 (1.06, 4.17)^a^
Targeted	Targeted-alone vs. targeted-plus	1.00 (0.24, 1.76)^a^	3.11 (0.64, 5.57)^a^
Targeted	Nontargeted vs. targeted-plus	0.16 (−0.59, 0.91)	0.49 (−1.91, 2.90)
Chemo + Immuno	Chemo + Immuno vs. Chemo-alone	1.02 (0.28, 1.76)^a^	2.98 (0.40, 5.56)^a^
Chemo + Immuno	Chemo + Immuno vs. IO-alone	0.09 (−1.21, 1.40)	−1.42 (−6.68, 3.85)
Chemo + Immuno	IO-alone vs. Chemo-alone	0.93 (−0.22, 2.09)	4.39 (−0.37, 9.16)
IO-alone	Chemo + Immuno vs. Chemo-alone	0.58 (−0.23, 1.39)	2.05 (−0.49, 4.59)

For each comparison, the 2L treatment associated with better 12-month survival is listed first.

a *P* < .05.

## Data Availability

The datasets analyzed for this study have been originated by Flatiron Health, Inc. Requests for data sharing by license or by permission for the specific purpose of replicating results in this manuscript can be submitted to dataaccess@flatiron.com.

## Clinical Practice Points

- Patients with aNSCLC progressing on first-line targeted therapy had longer survival when treated with a different single-agent targeted therapy in the second line, highlighting the potential benefit of sequential targeted approaches.
- Among patients previously treated with first-line chemo-immunotherapy or with slow-progressing disease treated with first-line immunotherapy, chemo-immunotherapy in the second line was associated with improved survival versus chemotherapy alone.
- These findings suggest that second-line treatment choice meaningfully impacts patient outcomes in aNSCLC and should be made based on prior treatment history, progression timing, and availability of targetable mutations.
- This study demonstrates the value of using real-world data from longitudinal electronic health records to assess comparative effectiveness where clinical trial evidence is limited or lacking.
- Prospective studies are needed to confirm these real-world findings and to better guide optimal second-line treatment strategies for patients with advanced NSCLC.
