# Supplementary material for: Real-World Effectiveness of Second-Line Therapies in Advanced Non-Small Cell Lung Cancer: Insights From Propensity-Weighted Comparative Analyses of Longitudinal EHR Data
Source: Clin Lung Cancer. Author manuscript; Available in PMC 2026 Apr 19. (PMC13092172; doi:10.1016/j.cllc.2025.09.010)
Supplement: 1 [file NIHMS2163466-supplement-1.pdf]

Supplemental Figures and Tables

Supplemental Figure 1 Line classification rules.

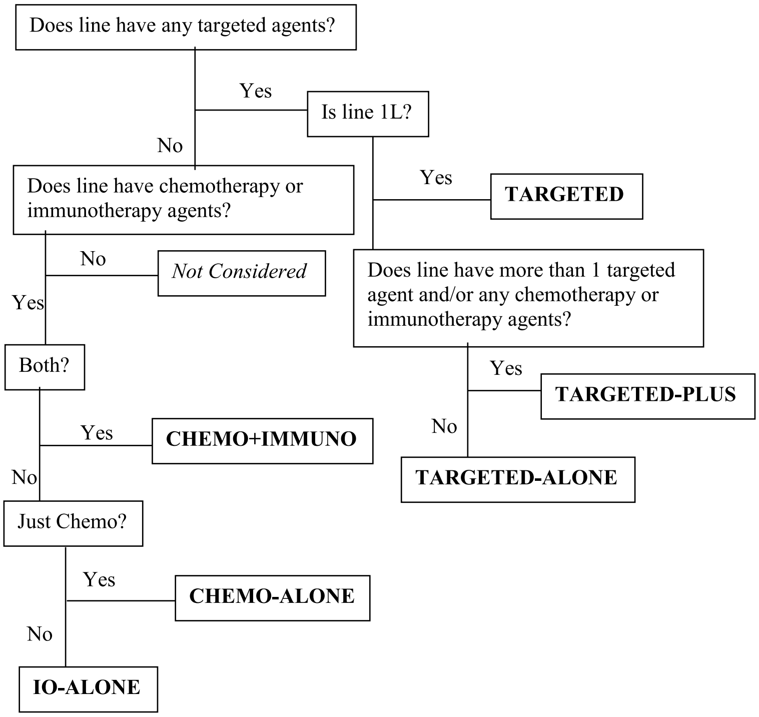

Supplemental Table 1 Number of patients on specific drug combinations in 1L and 2L. Only drug combinations received by 10 or more patients in either 1L or 2L in the comparison cohort are listed.

		Num. Patients	
**Line**	**2L Category**	**1L**	**2L**
Carboplatin, Pembrolizumab, Pemetrexed	Chemo+Immuno	793	302
Carboplatin, Paclitaxel, Pembrolizumab	Chemo+Immuno	142	69
Carboplatin, Paclitaxel Protein-Bound, Pembrolizumab	Chemo+Immuno	86	49
Abiraterone, Carboplatin, Pembrolizumab, Pemetrexed	Chemo+Immuno	22	0
Pembrolizumab, Pemetrexed	Chemo+Immuno	16	27
Atezolizumab, Bevacizumab, Carboplatin, Paclitaxel	Chemo+Immuno	11	16
Pembrolizumab	IO-alone	574	622
Nivolumab	IO-alone	356	2931
Atezolizumab	IO-alone	47	237
Durvalumab	IO-alone	45	11
Ipilimumab, Nivolumab	IO-alone	12	53
Erlotinib	Targeted-alone	779	25
Osimertinib	Targeted-alone	190	270
Afatinib	Targeted-alone	164	117
Crizotinib	Targeted-alone	156	10
Alectinib	Targeted-alone	43	44
Gefitinib	Targeted-alone	29	12
Dabrafenib, Trametinib	Targeted-plus	26	0
Cisplatin, Gemcitabine, Necitumumab	Targeted-plus	21	0
Docetaxel	Chemo-alone	0	834
Gemcitabine	Chemo-alone	0	671
Pemetrexed	Chemo-alone	0	630
Carboplatin, Pemetrexed	Chemo-alone	0	523
Docetaxel, Ramucirumab	Chemo-alone	0	483
Carboplatin, Paclitaxel	Chemo-alone	0	359
Carboplatin, Gemcitabine	Chemo-alone	0	288
Carboplatin, Paclitaxel Protein-Bound	Chemo-alone	0	235
Bevacizumab, Carboplatin, Pemetrexed	Chemo-alone	0	230
Vinorelbine	Chemo-alone	0	177
Bevacizumab, Carboplatin, Paclitaxel	Chemo-alone	0	173
Paclitaxel Protein-Bound	Chemo-alone	0	163
Bevacizumab, Pemetrexed	Chemo-alone	0	132
Paclitaxel	Chemo-alone	0	112
Carboplatin, Docetaxel	Chemo-alone	0	84
Cisplatin, Gemcitabine	Chemo-alone	0	63
Gemcitabine, Vinorelbine	Chemo-alone	0	57
Docetaxel, Gemcitabine	Chemo-alone	0	52
Bevacizumab, Docetaxel	Chemo-alone	0	51
Cisplatin, Pemetrexed	Chemo-alone	0	46
Carboplatin, Etoposide	Chemo-alone	0	35
Bevacizumab, Carboplatin, Paclitaxel Protein-Bound	Chemo-alone	0	30
Cisplatin, Etoposide	Chemo-alone	0	26
Bevacizumab	Chemo-alone	0	22
Bevacizumab, Carboplatin, Docetaxel	Chemo-alone	0	22
Bevacizumab, Gemcitabine	Chemo-alone	0	22
Bevacizumab-Awwb, Carboplatin, Pemetrexed	Chemo-alone	0	19
Bevacizumab, Paclitaxel Protein-Bound	Chemo-alone	0	17
Bevacizumab, Paclitaxel	Chemo-alone	0	16
Bevacizumab, Cisplatin, Pemetrexed	Chemo-alone	0	15

(*continued on next page*)

Supplemental Table 1 (*continued*)

Line	2L Category	Num. Patients	
		1L	2L
Topotecan	Chemo-alone	0	15
Carboplatin	Chemo-alone	0	14
Gemcitabine, Paclitaxel	Chemo-alone	0	14
Gemcitabine, Paclitaxel Protein-Bound	Chemo-alone	0	14
Bevacizumab, Carboplatin, Gemcitabine	Chemo-alone	0	11
Paclitaxel, Ramucirumab	Chemo-alone	0	10
Paclitaxel, Pembrolizumab	Chemo+Immuno	0	13
Carboplatin, Nivolumab, Paclitaxel	Chemo+Immuno	0	10
Gemcitabine, Pembrolizumab	Chemo+Immuno	0	10
Ceritinib	Targeted-alone	0	57
Lorlatinib	Targeted-alone	0	24
Brigatinib	Targeted-alone	0	11
Carboplatin, Erlotinib, Pemetrexed	Targeted-plus	0	22
Bevacizumab, Carboplatin, Erlotinib, Pemetrexed	Targeted-plus	0	10
Erlotinib, Pemetrexed	Targeted-plus	0	10

Supplemental Table 2A Specific drug combinations received in 2L broken down by 1L therapy category. Only drug combinations received by 20 or more patients in comparison cohort are listed.

2L Category	Line	Number of Patients by 1L		
		Targeted	Chemo+Immuno	IO-alone
Chemo-alone	Bevacizumab, Carboplatin, Paclitaxel	18	20	12
Chemo-alone	Bevacizumab, Carboplatin, Pemetrexed	69	12	44
Chemo-alone	Bevacizumab, Pemetrexed	7	3	14
Chemo-alone	Carboplatin, Docetaxel	9	5	7
Chemo-alone	Carboplatin, Gemcitabine	8	24	51
Chemo-alone	Carboplatin, Paclitaxel	29	53	55
Chemo-alone	Carboplatin, Paclitaxel Protein-Bound	15	22	51
Chemo-alone	Carboplatin, Pemetrexed	158	27	126
Chemo-alone	Docetaxel	24	110	58
Chemo-alone	Docetaxel, Gemcitabine	3	11	6
Chemo-alone	Docetaxel, Ramucirumab	9	281	50
Chemo-alone	Gemcitabine	27	84	75
Chemo-alone	Paclitaxel	6	14	8
Chemo-alone	Paclitaxel Protein-Bound	8	27	13
Chemo-alone	Pemetrexed	45	13	69
Chemo-alone	Vinorelbine	9	6	19
Chemo+Immuno	Carboplatin, Paclitaxel Protein-Bound, Pembrolizumab	2	6	15
Chemo+Immuno	Carboplatin, Paclitaxel, Pembrolizumab	1	11	29
Chemo+Immuno	Carboplatin, Pembrolizumab, Pemetrexed	90	14	87
Chemo+Immuno	Pembrolizumab, Pemetrexed	1	2	11
IO-alone	Nivolumab	122	21	0
IO-alone	Pembrolizumab	53	36	0
Targeted-alone	Afatinib	117	0	0
Targeted-alone	Alectinib	44	0	0
Targeted-alone	Ceritinib	57	0	0
Targeted-alone	Erlotinib	25	0	0
Targeted-alone	Lorlatinib	24	0	0
Targeted-alone	Osimertinib	270	0	0
Targeted-plus	Carboplatin, Erlotinib, Pemetrexed	22	0	0

Supplemental Table 2B Specific 1L-2L drug combinations received by 10 or more patients in comparison cohort.

1L Category	2L Category	1L Line	2L Line	Num. Patients
Chemo+Immuno	Chemo-alone	Carboplatin, Pembrolizumab, Pemetrexed	Docetaxel, Ramucirumab	217
Chemo+Immuno	Chemo-alone	Carboplatin, Pembrolizumab, Pemetrexed	Docetaxel	92
Chemo+Immuno	Chemo-alone	Carboplatin, Pembrolizumab, Pemetrexed	Carboplatin, Paclitaxel	38
Chemo+Immuno	Chemo-alone	Carboplatin, Pembrolizumab, Pemetrexed	Gemcitabine	36
Chemo+Immuno	Chemo-alone	Carboplatin, Paclitaxel, Pembrolizumab	Docetaxel, Ramucirumab	27
Chemo+Immuno	Chemo-alone	Carboplatin, Pembrolizumab, Pemetrexed	Paclitaxel Protein-Bound	24
Chemo+Immuno	Chemo-alone	Carboplatin, Paclitaxel, Pembrolizumab	Gemcitabine	22
Chemo+Immuno	Chemo-alone	Carboplatin, Pembrolizumab, Pemetrexed	Carboplatin, Pemetrexed	20
Chemo+Immuno	Chemo-alone	Carboplatin, Paclitaxel Protein-Bound, Pembrolizumab	Docetaxel, Ramucirumab	18
Chemo+Immuno	Chemo-alone	Carboplatin, Paclitaxel Protein-Bound, Pembrolizumab	Gemcitabine	17
Chemo+Immuno	Chemo-alone	Carboplatin, Pembrolizumab, Pemetrexed	Bevacizumab, Carboplatin, Paclitaxel	17
Chemo+Immuno	Chemo-alone	Carboplatin, Pembrolizumab, Pemetrexed	Paclitaxel	14
Chemo+Immuno	Chemo-alone	Carboplatin, Pembrolizumab, Pemetrexed	Carboplatin, Paclitaxel Protein-Bound	13
Chemo+Immuno	Chemo-alone	Carboplatin, Pembrolizumab, Pemetrexed	Bevacizumab, Carboplatin, Pemetrexed	11
Chemo+Immuno	IO-alone	Carboplatin, Pembrolizumab, Pemetrexed	Pembrolizumab	24
Chemo+Immuno	IO-alone	Carboplatin, Pembrolizumab, Pemetrexed	Nivolumab	15
IO-alone	Chemo-alone	Pembrolizumab	Carboplatin, Pemetrexed	101
IO-alone	Chemo-alone	Nivolumab	Gemcitabine	44
IO-alone	Chemo-alone	Nivolumab	Docetaxel, Ramucirumab	39
IO-alone	Chemo-alone	Pembrolizumab	Carboplatin, Paclitaxel	38
IO-alone	Chemo-alone	Pembrolizumab	Pemetrexed	36
IO-alone	Chemo-alone	Nivolumab	Docetaxel	35
IO-alone	Chemo-alone	Pembrolizumab	Bevacizumab, Carboplatin, Pemetrexed	34
IO-alone	Chemo-alone	Pembrolizumab	Carboplatin, Paclitaxel Protein-Bound	33
IO-alone	Chemo-alone	Pembrolizumab	Carboplatin, Gemcitabine	31
IO-alone	Chemo-alone	Nivolumab	Pemetrexed	25
IO-alone	Chemo-alone	Nivolumab	Carboplatin, Pemetrexed	21
IO-alone	Chemo-alone	Pembrolizumab	Gemcitabine	19
IO-alone	Chemo-alone	Pembrolizumab	Docetaxel	17
IO-alone	Chemo-alone	Nivolumab	Carboplatin, Gemcitabine	16
IO-alone	Chemo-alone	Nivolumab	Carboplatin, Paclitaxel Protein-Bound	16
IO-alone	Chemo-alone	Nivolumab	Carboplatin, Paclitaxel	12
IO-alone	Chemo-alone	Nivolumab	Vinorelbine	11
IO-alone	Chemo+Immuno	Pembrolizumab	Carboplatin, Pembrolizumab, Pemetrexed	81
IO-alone	Chemo+Immuno	Pembrolizumab	Carboplatin, Paclitaxel, Pembrolizumab	26
IO-alone	Chemo+Immuno	Pembrolizumab	Carboplatin, Paclitaxel Protein-Bound, Pembrolizumab	13
Targeted	Chemo-alone	Erlotinib	Carboplatin, Pemetrexed	106
Targeted	Chemo-alone	Erlotinib	Bevacizumab, Carboplatin, Pemetrexed	46
Targeted	Chemo-alone	Erlotinib	Pemetrexed	39
Targeted	Chemo-alone	Osimertinib	Carboplatin, Pemetrexed	25
Targeted	Chemo-alone	Erlotinib	Docetaxel	17
Targeted	Chemo-alone	Erlotinib	Gemcitabine	17
Targeted	Chemo-alone	Erlotinib	Carboplatin, Paclitaxel	16
Targeted	Chemo-alone	Erlotinib	Bevacizumab, Carboplatin, Paclitaxel	15
Targeted	Chemo-alone	Crizotinib	Carboplatin, Pemetrexed	13
Targeted	Chemo+Immuno	Osimertinib	Carboplatin, Pembrolizumab, Pemetrexed	37
Targeted	Chemo+Immuno	Afatinib	Carboplatin, Pembrolizumab, Pemetrexed	13

(*continued on next page*)

Supplemental Table 2B (*continued*)

1L Category	2L Category	1L Line	2L Line	Num. Patients
Targeted	Chemo+Immuno	Dabrafenib, Trametinib	Carboplatin, Pembrolizumab, Pemetrexed	12
Targeted	Chemo+Immuno	Erlotinib	Carboplatin, Pembrolizumab, Pemetrexed	11
Targeted	IO-alone	Erlotinib	Nivolumab	62
Targeted	IO-alone	Osimertinib	Pembrolizumab	18
Targeted	IO-alone	Afatinib	Nivolumab	13
Targeted	IO-alone	Afatinib	Pembrolizumab	11
Targeted	IO-alone	Cisplatin, Gemcitabine, Necitumumab	Nivolumab	11
Targeted	Targeted-alone	Erlotinib	Osimertinib	170
Targeted	Targeted-alone	Erlotinib	Afatinib	104
Targeted	Targeted-alone	Afatinib	Osimertinib	73
Targeted	Targeted-alone	Crizotinib	Ceritinib	52
Targeted	Targeted-alone	Crizotinib	Alectinib	42
Targeted	Targeted-alone	Gefitinib	Osimertinib	19
Targeted	Targeted-alone	Alectinib	Lorlatinib	14
Targeted	Targeted-plus	Erlotinib	Carboplatin, Erlotinib, Pemetrexed	22

Supplemental Table 3 Variables included in additional confounder sets. The Extended set augments the main confounder set with the listed variables. The Comprehensive set augments the Extended set with the additional listed variables. The Comprehensive set was also used for imputation models.

Set	Variable
Extended	Time between first and last 1L medication administration
Extended	Time from 1L progression to 2L initiation
Extended	Body Mass Index (BMI) at 2L initiation
Extended	Practice type (Academic/Community)
Extended	Number of recorded visits before 2L initiation
Extended	Patient Region (Midwest/Northeast/South/West)
Extended	Number of recorded progressions while on 1L
Extended	Hemoglobin at 2L initiation
Extended	Change in Hemoglobin between 1L and 2L
Extended	Creatinine at 2L initiation
Extended	Change in Creatinine between 1L and 2L
Extended	Bilirubin at 2L initiation
Extended	Change in Bilirubin between 1L and 2L
Extended	Albumin at 2L initiation
Extended	Change in Albumin between 1L and 2L
Extended	Change in Body Weight between 1L and 2L
Comprehensive	Neutrophil count at 2L initiation
Comprehensive	Change in Neutrophil count between 1L and 2L
Comprehensive	Glucose at 2L initiation
Comprehensive	Change in Glucose between 1L and 2L
Comprehensive	Protein at 2L initiation
Comprehensive	Change in Protein between 1L and 2L
Comprehensive	Platelets at 2L initiation
Comprehensive	Change in Platelets between 1L and 2L
Comprehensive	Proportion of patients at practice given Targeted regimen in 1L
Comprehensive	Proportion of patients at practice given Chemo-alone regimen in 1L
Comprehensive	Proportion of patients at practice given same regimen in 1L/2L

Supplemental Figure 2 Proportion of patients initiating 2L each year receiving a particular regimen in overall cohort.

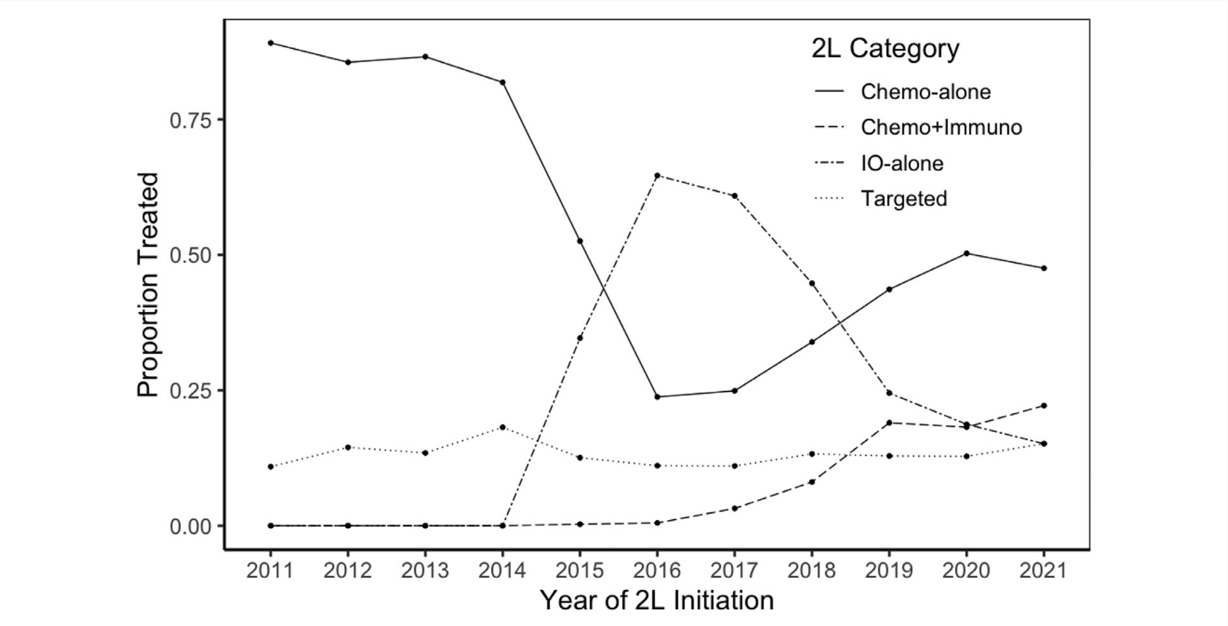

Supplemental Figure 3 Subgroup analyses comparing effects of 2L treatment choice on rwOS RMST at 36 months when enough data is available for a given subgroup. The 2L option with better RMST-36 on the full cohort is always listed first.

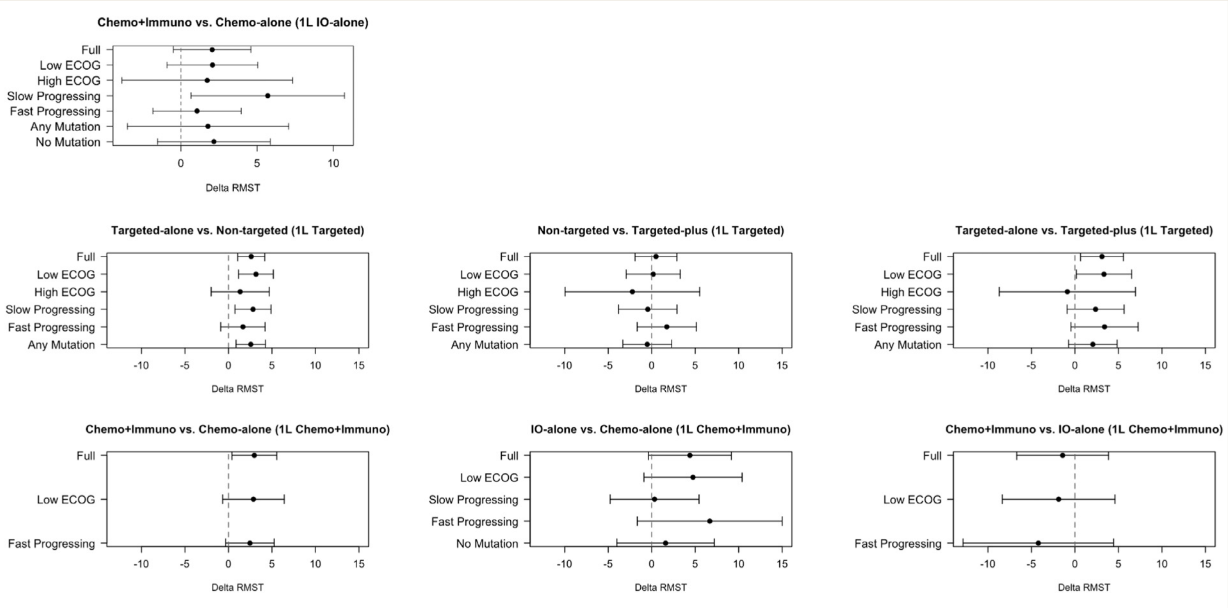

Supplemental Figure 4 IPW-Adjusted Kaplan Meier curves for rwPFS stratified by 2L treatment choice.

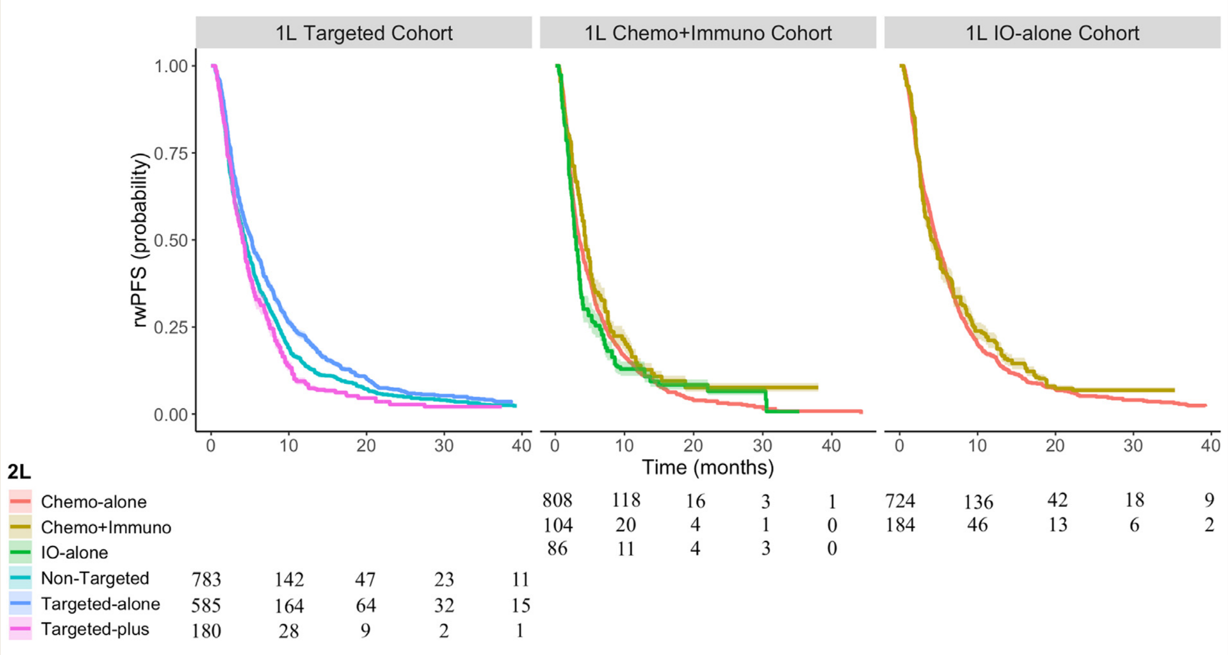

Supplemental Figure 5 IPW-adjusted rwPFS summaries for 1L Targeted cohort by 2L treatment choice and estimated differences in RMSTs between 2L options. The 2L group with better overall survival is always listed first in comparisons. (*p < 0.05)

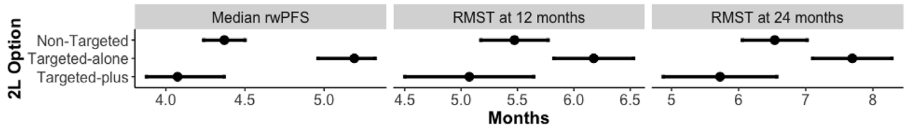

ΔRMST-12 (months)	ΔRMST-24 (months)
Targeted-alone vs. Non-Targeted 0.70 (0.24, 1.17)*	Targeted-alone vs. Non-Targeted 1.16 (0.39, 1.92)*
Targeted-alone vs. Targeted-plus 1.10 (0.43, 1.78)*	Targeted-alone vs. Targeted-plus 1.97 (0.94, 3.00)*
Non-targeted vs. Targeted-plus 0.40 (-0.25, 1.05)	Non-targeted vs. Targeted-plus 0.81 (-0.16, 1.79)

Supplemental Figure 6 IPW-adjusted rwPFS summaries for 1L Chemo+Immuno cohort by 2L treatment choice and estimated differences in RMSTs between 2L options. The 2L group with better overall survival is always listed first in comparisons. (*p < 0.05)

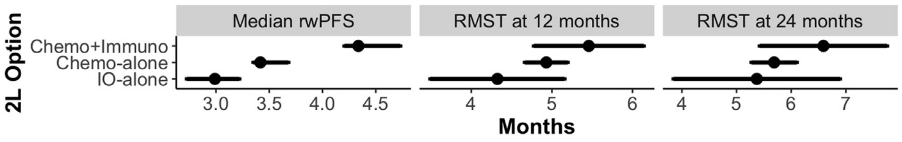

ΔRMST-12 (months)	ΔRMST-24 (months)
Chemo+Immuno vs. Chemo-alone	Chemo+Immuno vs. Chemo-alone
0.53 (-0.20, 1.26)	0.90 (-0.33, 2.14)
Chemo+Immuno vs. IO-alone	Chemo+Immuno vs. IO-alone
1.13 (0.06, 2.21)*	1.22 (-0.70, 3.14)
Chemo-alone vs. IO-alone	Chemo-alone vs. IO-alone
0.61 (-0.27, 1.48)	0.32 (-1.26, 1.90)

Supplemental Figure 7 IPW-adjusted rwPFS summaries for 1L IO-alone cohort by 2L treatment choice and estimated differences in RMSTs between 2L options. The 2L group with better overall survival is always listed first in comparisons. (*p < 0.05)

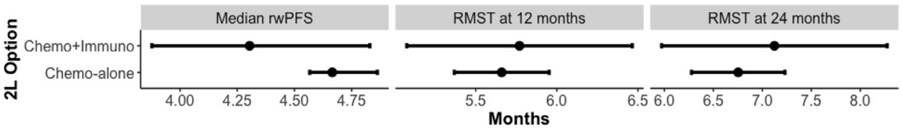

ΔRMST-12 (months)	ΔRMST-24 (months)
Chemo+Immuno vs. Chemo-alone	Chemo+Immuno vs. Chemo-alone
0.11 (-0.64, 0.86)	0.37 (-0.88, 1.62)

Supplemental Table 4 Characteristics of patients receiving 1L Targeted stratified by 2L receipt in unweighted cohort.

Patient Characteristic	2L Non-Targeted (n=783)	2L Targeted-Alone (n=585)	2L Targeted-Plus (n=180)
Advanced Stage at Initial Diagnosis, No. (%)	578 (73.8)	475 (81.2)	153 (85.0)
Age at 2L Initiation, Mean (SD)	67.53 (10.04)	66.31 (11.68)	65.68 (10.00)
Change in ECOG score during 1L, Mean (SD)	0.20 (0.81)	0.09 (0.78)	0.01 (0.82)
ECOG score at 2L Initiation, No. (%)			
0	143 (18.3)	147 (25.1)	52 (28.9)
1	301 (38.4)	203 (34.7)	71 (39.4)
2-3	147 (18.8)	91 (15.6)	22 (12.2)
Unknown	192 (24.5)	144 (24.6)	35 (19.4)
Insurance Type, No. (%)			
Commercial	177 (22.6)	170 (29.1)	56 (31.1)
Ever on Medicaid	78 (10.0)	61 (10.4)	18 (10.0)
Government	289 (36.9)	220 (37.6)	70 (38.9)
Other	144 (18.4)	91 (15.6)	24 (13.3)
Unknown	95 (12.1)	43 (7.4)	12 (6.7)
Male, No. (%)	313 (40.0)	212 (36.2)	63 (35.0)
Number of new diagnoses during 1L, Mean (SD)	0.69 (0.99)	0.79 (1.15)	0.77 (0.97)
Smoking History, No. (%)			
No	312 (39.8)	326 (55.7)	99 (55.0)
Yes	460 (58.7)	258 (44.1)	80 (44.4)
Unknown	11 (1.4)	1 (0.2)	1 (0.6)
Time Until Progression on 1L, Mean (SD)	6.99 (5.18)	9.32 (5.41)	7.95 (5.01)
Treated at Community Practice, No. (%)	693 (88.5)	463 (79.1)	134 (74.4)
White, No. (%)			
No	245 (31.3)	187 (32.0)	62 (34.4)
Yes	488 (62.3)	359 (61.4)	108 (60.0)
Unknown	50 (6.4)	39 (6.7)	10 (5.6)
Year of Advanced Stage Diagnosis, No. (%)			
<2016	444 (56.7)	263 (45.0)	90 (50.0)
2016-2019	258 (33.0)	282 (48.2)	55 (30.6)
2019+	81 (10.3)	40 (6.8)	35 (19.4)

Supplemental Table 5 Characteristics of patients receiving 1L Chemo+Immuno stratified by 2L receipt in unweighted cohort.

Patient Characteristic	2L Chemo-alone (n=808)	2L Chemo+Immuno (n=104)	2L IO-alone (n=86)
Advanced Stage at Initial Diagnosis, No. (%)	653 (80.8)	88 (84.6)	70 (81.4)
Age at 2L Initiation, Mean (SD)	66.86 (9.18)	66.74 (9.11)	69.60 (8.75)
Change in ECOG score during 1L, Mean (SD)	0.23 (0.70)	0.18 (0.66)	0.10 (0.64)
ECOG score at 2L Initiation, No. (%)			
0	188 (23.3)	34 (32.7)	20 (23.3)
1	364 (45.0)	39 (37.5)	34 (39.5)
2-3	169 (20.9)	22 (21.2)	18 (20.9)
Unknown	87 (10.8)	9 (8.7)	14 (16.3)
Insurance Type, No. (%)			
Commercial	216 (26.7)	20 (19.2)	18 (20.9)
Ever on Medicaid	98 (12.1)	14 (13.5)	15 (17.4)
Government	298 (36.9)	41 (39.4)	32 (37.2)
Other	155 (19.2)	26 (25.0)	17 (19.8)
Unknown	41 (5.1)	3 (2.9)	4 (4.7)
Male, No. (%)	457 (56.6)	52 (50.0)	45 (52.3)
Number of new diagnoses during 1L, Mean (SD)	0.85 (1.16)	0.94 (1.01)	1.12 (1.42)
Smoking History, No. (%)	734 (90.8)	93 (89.4)	80 (93.0)
Squamous Histology at Initial Diagnosis, No. (%)			
No	632 (78.2)	75 (72.1)	67 (77.9)
Yes	150 (18.6)	22 (21.2)	16 (18.6)
Unknown	26 (3.2)	7 (6.7)	3 (3.5)
Time Until Progression on 1L, Mean (SD)	6.11 (4.45)	5.75 (8.69)	8.69 (6.19)
White, No. (%)			
No	206 (25.5)	29 (27.9)	22 (25.6)
Yes	552 (68.3)	70 (67.3)	60 (69.8)
Unknown	50 (6.2)	5 (4.8)	4 (4.7)
Year of Advanced Stage Diagnosis Before 2019, No. (%)	378 (46.8)	46 (44.2)	46 (53.5)

Supplemental Table 6 Characteristics of patients receiving 1L IO-alone stratified by 2L receipt in unweighted cohort.

Patient Characteristic	2L Chemo+Immuno (n=184)	2L Chemo-alone (n=724)
Advanced Stage at Initial Diagnosis, No. (%)	121 (65.8)	326 (45.0)
Age at 2L Initiation, Mean (SD)	70.02 (9.27)	69.35 (9.54)
Change in ECOG score during 1L, Mean (SD)	0.21 (0.70)	0.23 (0.72)
ECOG score at 2L Initiation, No. (%)		
0	49 (26.6)	141 (19.5)
1	77 (41.8)	333 (46.0)
2-3	40 (21.7)	150 (20.7)
Unknown	18 (9.8)	100 (13.8)
Insurance Type, No. (%)		
Commercial	49 (26.6)	148 (20.4)
Ever on Medicaid	24 (13.0)	86 (11.9)
Government	78 (42.4)	310 (42.8)
Other	30 (16.3)	147 (20.3)
Unknown	3 (1.6)	33 (4.6)
Male, No. (%)	86 (46.7)	378 (52.2)
Number of new diagnoses during 1L, Mean (SD)	0.71 (1.01)	0.60 (0.90)
Smoking History, No. (%)	165 (89.7)	670 (92.5)
Squamous Histology at Initial Diagnosis, No. (%)		
No	127 (69.0)	436 (60.2)
Yes	48 (26.1)	265 (36.6)
Unknown	9 (4.9)	23 (3.2)
Time Until Progression on 1L, Mean (SD)	5.09 (5.17)	5.41 (4.64)
Treated at Community Practice, No. (%)	155 (84.2)	657 (90.7)
White (%)		
No	45 (24.5)	163 (22.5)
Yes	129 (70.1)	522 (72.1)
Unknown	10 (5.4)	39 (5.4)
Year of Advanced Stage Diagnosis Before 2019, No. (%)	104 (56.5)	583 (80.5)

Supplemental Table 7 Balancing metrics by weighting approach.

1L Treatment	Weight Model	Variable Set	Mean SMD	Max SMD	% Covariates Well-Balanced (SMD < 0.1)
Targeted	Logistic Regression	Primary	0.03	0.07	100
	Energy	Extended	0.02	0.07	100
	LASSO	Comprehensive	0.05	0.15	93
Chemo+Immuno	Logistic Regression	Primary	0.06	0.15	96
	Energy	Extended	0.05	0.14	93
	LASSO	Comprehensive	0.09	0.25	60
IO-alone	Logistic Regression	Primary	0.04	0.09	100
	Energy	Extended	0.01	0.05	100
	LASSO	Comprehensive	0.07	0.25	73

Supplemental Table 8 Comparison of estimates for difference in rwOS RMST between 2L options by exclusion period (14 or 28 days) and weighting approach for patients receiving 1L Targeted. The 2L option with better 12-month RMST in the main analysis is always listed first. (*p-value < 0.05)

2L Comparison	Weights	Excl. Period	ΔRMST-12 (months)	ΔRMST-36 (months)
Targeted-alone vs. Non-Targeted	Logistic Regression	14	0.84 (0.36, 1.31)*	2.61 (1.06, 4.17)*
	Logistic Regression	28	0.81 (0.36, 1.28)*	2.60 (1.03, 4.16)*
	Unweighted	14	1.21 (0.81, 1.62)*	3.95 (2.60, 5.30)*
	Energy + Extended	14	0.82 (0.31, 1.34)*	2.27 (0.58, 3.96)*
	LASSO + Comprehensive	14	0.84 (0.38, 1.31)*	2.68 (1.15, 4.22)*
Non-Targeted vs. Targeted-plus	Logistic Regression	14	0.16 (-0.59, 0.91)	0.49 (-1.91, 2.90)
	Logistic Regression	28	0.08 (-0.65, 0.82)	0.38 (-2.06, 2.82)
	Unweighted	14	0.32 (-0.32, 0.96)	1.18 (-0.92, 3.28)
	Energy + Extended	14	0.27 (-0.50, 1.04)	1.03 (-1.46, 3.52)
	LASSO + Comprehensive	14	0.29 (-0.55, 1.13)	0.47 (-2.24, 3.17)
Targeted-alone vs. Targeted-plus	Logistic Regression	14	1.00 (0.24, 1.76)*	3.11 (0.64, 5.57)*
	Logistic Regression	28	0.90 (0.16, 1.64)*	2.98 (0.48, 5.47)*
	Unweighted	14	0.89 (0.25, 1.54)*	2.77 (0.59, 4.94)*
	Energy + Extended	14	1.09 (0.31, 1.88)*	3.30 (0.74, 5.86)*
	LASSO + Comprehensive	14	1.13 (0.28, 1.98)*	3.15 (0.39, 5.91)*

Supplemental Table 9 Comparison of estimates for difference in rwOS RMST between 2L options by exclusion period and weighting approach for patients receiving 1L Chemo+Immuno. The 2L option with better 12-month RMST in the main analysis is always listed first. (*p-value < 0.05)

2L Comparison	Weights	Excl. Period	ΔRMST-12 (months)	ΔRMST-36 (months)
Chemo+Immuno vs. Chemo-alone	Logistic Regression	14	1.02 (0.28, 1.76)*	2.98 (0.40, 5.56)*
	Logistic Regression	28	1.02 (0.31, 1.73)*	3.05 (0.42, 5.69)*
	Unweighted	14	1.04 (0.23, 1.87)*	3.07 (0.48, 5.66)*
	Energy + Extended	14	0.47 (-0.34, 1.28)	2.19 (-0.76, 5.14)
	LASSO + Comprehensive	14	0.79 (0.04, 1.54)*	2.80 (0.08, 5.52)*
Chemo+Immuno vs. IO-alone	Logistic Regression	14	0.09 (-1.21, 1.40)	-1.42 (-6.68, 3.85)
	Logistic Regression	28	0.06 (-1.23, 1.35)	-1.56 (-7.08, 3.96)
	Unweighted	14	-0.29 (-1.45, 0.87)	-1.21 (-5.00, 2.58)
	Energy + Extended	14	-0.23 (-1.52, 1.07)	-0.87 (5.96, 4.22)
	LASSO + Comprehensive	14	-0.24 (-1.54, 1.06)	-0.55 (-5.61, 4.51)
IO-alone vs. Chemo-alone	Logistic Regression	14	0.93 (-0.22, 2.09)	4.39 (-0.37, 9.16)
	Logistic Regression	28	0.96 (-0.19, 2.12)	4.61 (-0.40, 9.63)
	Unweighted	14	1.33 (0.41, 2.25)*	4.27 (1.31, 7.25)*
	Energy + Extended	14	0.70 (-0.43, 1.82)	3.06 (-1.37, 7.49)
	LASSO + Comprehensive	14	0.98 (-0.07, 2.04)	3.72 (-0.41, 7.85)

Supplemental Table 10 Comparison of estimates for difference in rwOS RMST between 2L options by exclusion period and weighting approach for patients receiving 1L IO-alone. The 2L option with better 12-month RMST in the main analysis is always listed first. (*p-value < 0.05)

2L Comparison	Weights	Excl. Period	ΔRMST-12 (months)	ΔRMST-36 (months)
Chemo+Immuno vs. Chemo-alone	Logistic Regression	14	0.58 (-0.23, 1.39)	2.05 (-0.49, 4.59)
	Logistic Regression	28	0.55 (-0.24, 1.34)	2.11 (-0.46, 4.67)
	Unweighted	14	0.63 (-0.04, 1.30)	2.54 (0.50, 4.58)*
	Energy + Extended	14	0.49 (-0.31, 1.29)	1.81 (-0.72, 4.33)
	LASSO + Comprehensive	14	0.62 (-0.15, 1.40)	2.36 (-0.15, 4.87)
